# Antagonistic effect of dopamine structural analogues on human GABAρ1 receptor

**DOI:** 10.1038/s41598-017-17530-8

**Published:** 2017-12-12

**Authors:** Alfredo Alaniz-Palacios, Ataulfo Martínez-Torres

**Affiliations:** Departamento de Neurobiología Celular y Molecular, Laboratorio de Neurobiología Molecular y Celular, Instituto de Neurobiología, Universidad Nacional Autónoma de México, Juriquilla, 76230 Santiago de Querétaro, Querétaro, Mexico

## Abstract

GABAergic and dopaminergic pathways are co-localized in several areas of the central nervous system and recently several reports have shown co-release of both neurotransmitters. The GABA-A receptor (β and ρ1 subunits) is modulated by dopamine (DA) and, interestingly, GABAρ1 can be modulated by several biogenic amines. Here we explored the effects of the metabolites of the dopaminergic pathway and other structural analogues of DA on GABAρ1 and the DA gated ion channel (LGC-53) from *Caenorhabditis elegans* expressed in *Xenopus laevis* oocytes. Our findings show an antagonistic effect of the metabolite 3-Methoxytyramine (3-MT, IC_50_ = 285 ± 30 µM) with similar potency compared to DA on induced GABA currents; however, it was inactive on LGC-53. The structural DA analogues and metabolites, 3, 4-dihydroxyphenylacetic acid (DOPAC), homovanillic acid (HVA), 2-phenylethylamine (β-PEA) and 4-amino-1-butanol (4-AM-1-OH), antagonized GABAρ1 currents, whereas β-PEA acted as partial agonists on LGC-53, indicating that the putative binding sites of both receptors may share structural characteristics. These results suggest that the DA metabolites 3-MT, DOPAC and HVA modulate GABAρ1 and possibly affect the activity of the receptors that include this subunit *in vivo*.

## Introduction

γ-Aminobutyric acid (GABA) is the most abundant inhibitory neurotransmitter in the central nervous system (CNS), and its co-localization with other neurotransmitter pathways has been fully demonstrated. Interestingly, the co-release of GABA and dopamine (DA) has been reported in the striatum and retina^1,2^; however, the physiological consequences of this convergence are not fully understood. There are two main components that give rise to the ionotropic GABA responses. The first component is the most abundant GABA-A receptor widely distributed throughout the nervous system and effectively blocked by the alkaloid bicuculline. This receptor is formed by a combination of α, β and γ subunits and by other less abundant subunits (δ, θ, ε, π)^3^. The second component does not desensitize after prolonged exposure to the agonist, is insensitive to bicuculline^4,5^ and the (1,2,5,6-Tetrahydropyridin-4-yl) methylphosphinic acid (TPMPA) is a competitive antagonist^6^; is abundantly expressed in retina and has also been found in several areas of the brain; for example, the cerebellum^7^, hippocampus^8^ and striatum^9^. This GABA receptor is formed by ρ subunits (ρ1–ρ3) and is commonly known as GABA-C^10,11^. Today we know that the three GABAρ subunits are phylogenetically related to classic GABA-A subunits (α, β, γ) included in the Cys-loop family of neurotransmitter receptors, which form pentameric assemblies and gate a chloride channel upon activation. In addition, ρ1 subunits assemble with classic GABA-A subunits and form heteromeric complexes with different characteristics^12–14^.

One of the special characteristics of the human GABAρ1 receptor reported in a previous communication showed a negative modulation by monoamines such as DA, serotonin and tyramine^15^. The GABAρ1 subunit cloned from *Sus scrofa* ^16^ is also modulated by DA, thus, the effect does not seem to be species specific. More recently, it was found that GABA-A receptors from striatal neurons are efficiently gated by DA; however, the effect is dependent on the presence of the GABA-A β3 subunit^17^. Another example of cross-talk between the GABAergic system and other neurotransmitters involves the allosteric potentiation of ATP on GABA-A receptors^18^. This interaction is not exclusive to the GABAergic system, since similar effects have been described in the past between neurotransmitters such as the serotoninergic modulation of nicotinic receptors^19,20^.

The Cys-loop family of neurotransmitter receptors includes several ionotropic DA receptors, such as LGC-53, isolated from *Caenorhabditis elegans* ^21^, and other DA receptors known from invertebrates^22,23^. These findings suggest that during the evolution of the Cys-loop family of receptors, the mutations that increased sensitivity and specificity for an agonist^24^ did not necessarily fully eliminate the recognition for other ligands. The presence of a second ligand-binding site makes it possible to diversify the neurotransmitter signaling and would allow the receptor to be targeted not only by the second neurotransmitter but also by structurally similar molecules.

LGC-53 is activated by DA but not by serotonin (5HT), octopamine, tyramine or histamine, yet it is sensitive to modulators of mammalian metabotropic DA receptors such as haloperidol, risperidone and spiperone^21^. LGC-53 has several characteristics in common with GABA-A receptors^17^, and in particular with GABAρ1. For example, it is permeable to Cl^−^ and forms functional homopentamers when expressed in heterologous systems. These characteristics make this receptor suitable for comparative functional and structural studies.

In this communication, we report the negative modulation of GABAρ1 by DA metabolites: 3-methoxytyramine (3-MT), 3, 4-dihydroxyphenylacetic acid (DOPAC), homovanillic acid (HVA), and structural DA analogues 2-phenylethylamine (β-PEA) and 4-amino-1-butanol (4-AM-1-OH). In addition, we tested whether these compounds gate the inotropic DA receptor LGC-53. Our findings extend the list of compounds that act as modulators of GABAρ1 and suggest that these molecules may execute some effect in the GABAergic system *in vivo*, where they are commonly found (e.g., the brain^25^ and retina^26^).

## Results

Oocytes injected with ρ1 generated non-desensitizing ionic currents upon perfusion of GABA, whereas those injected with LGC-53 generated fast, desensitizing ionic currents when DA was perfused. Currents showing run-down or run-up (>20% of change) during three consecutive applications were discarded. Non-injected oocytes and oocytes injected with human ρ1 did not generate evident currents when exposed to up to 1 mM DA.

**Figure 1:**
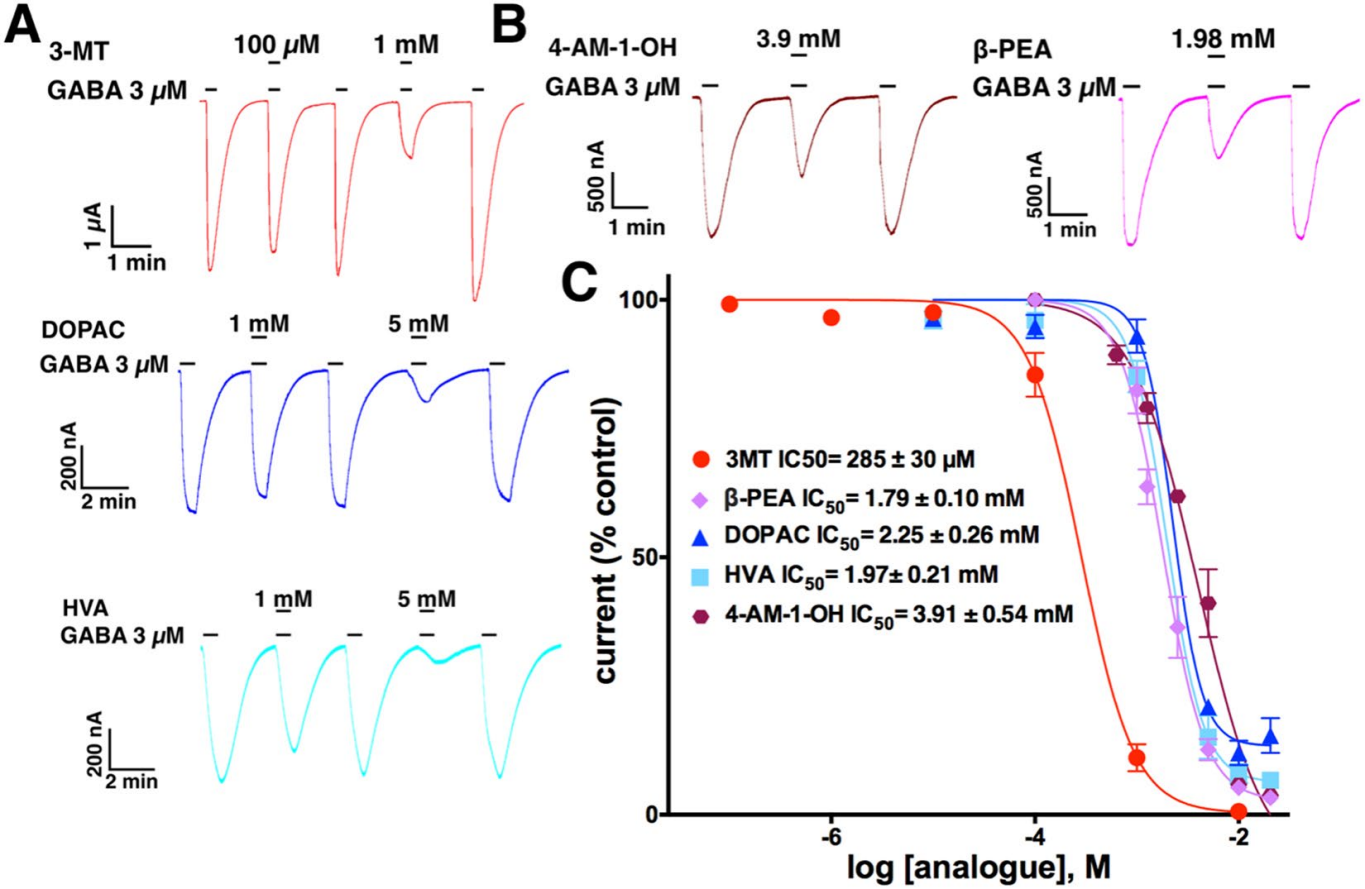
Effect of DA analogues on GABA currents. (**A**) Sample currents of co-applications of 3 µM GABA and DA metabolites. (**B**) Sample currents of co-applications of 3 µM GABA and a DA analogues. (**C**) Concentration-response relations. IC_50_ for each compound is shown in inset. Data were normalized to the response to 3 µM GABA. 3-MT shows a similar potency to DA (previously reported by Ochoa de la Paz, 2012). Each point was evaluated in 6 oocytes from 3 frogs.

GABA currents were negatively and concentration-dependently modulated by DA metabolites like 3-MT (IC_50_ = 285 ± 30 µM, R^2^ = 0.986), DOPAC (IC_50_ = 2.25 ± 0.26 mM, R^2^ = 0.977) and HVA (IC_50_ = 1.97 ± 0.20 mM, R^2^ = 0.976). Representative recordings and concentration-response curves are shown in Fig. 1A–C (Eq. 1, 2 and 3). Other DA analogues modulated the GABA currents negatively (Fig. 1B–C) but were less potent; for example, 4-AM-1-OH (IC_50_ = 3.91 ± 0.54 mM, R^2^ = 0.978) and β-PEA (IC_50_ = 1.79 ± 0.10 mM, R^2^ = 0.957). This result shows that 3-MT inhibits the GABA currents with similar potency to DA and is more potent than other previously tested biogenic amines such as 5 hydroxy-tryptamine, tyramine and octopamine^15^. L-DOPA, adrenaline (AD) and noradrenaline (NAD) were evaluated at concentrations up to 20 mM and failed to elicit any effect on the responses generated by 3 μM GABA (data not shown). To determine that the inhibitory effect is not due to the oocyte expression system, we evaluated whether DA and 3-MT inhibit GABA responses of GABAρ1 expressed in HEK cells. As shown in Supplementary Fig. S1, the inhibitory effect was similar to that in oocytes.

**Figure 2:**
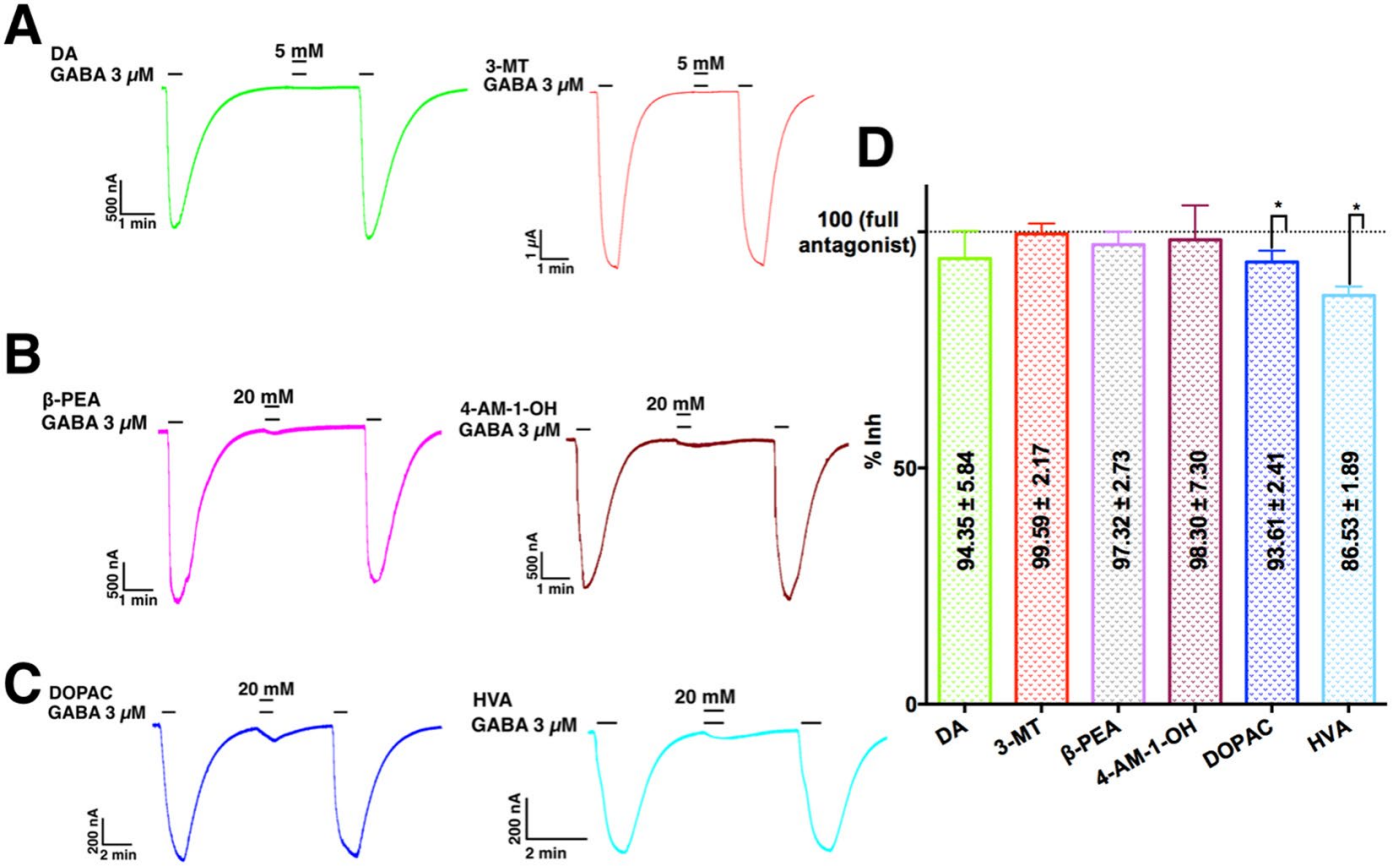
Maximal effect of DA analogues on GABA currents. Only amines for (**A)** Sample currents of co-applications of 3 µM GABA and DA or 3-MT; amine moiety with current remnant for (**B**) Sample currents of co-applications of 3 µM GABA and β-PEA or 4-AM-1-OH. Acid moiety. (**C**) Sample currents of co-applications of 3 µM GABA and/or HVA. (**D**) Maximal effect of the analogues. Mean and SEM are indicated inside the corresponding bar. Only HVA and DOPAC did not fully inhibit GABA-induced currents. Data are from to 6 cells from 3 frogs.

Full inhibition of GABA currents was observed at high concentrations of 3-MT and DA (Fig. 2A), while the rest of the compounds did not fully block the GABA currents and consistently showed residual responses (Fig. 2B and C), suggesting a partial antagonism^27^. Data obtained from the concentration-response curves at the maximum inhibition was compared with the full inhibition (100%, Eq. 3) for each compound. The comparison revealed that DOPAC and HVA, which has an acidic moiety, was significantly different from the full inhibition (Fig. 2D). The summary (mean and SEM) of maximal effects of the analogues from concentration-response curves are indicated in the corresponding bar in Fig. 2D.

All these compounds are ionized at pH 7.4. In order to determine the effect of the ligand charge on the antagonist-receptor interaction, a current-voltage ramp protocol was performed from −120 to +70 mV in presence of the IC_50_ concentration for each compound (n = 6 oocytes, Supplementary Fig. 2). The results obtained did not show significant differences in their inhibitory effects on the ramp voltage, indicating that the formation of the receptor-compound complex is insensitive to the voltage and suggesting an independent mechanism of the pore-blockage inhibition in agreement with previous reports for DA^15^. Additionally, the reversal potential observed here for GABA currents was consistent with other reports^28,29^. This reversal potential did not change in presence of these compounds (−35.25 ± 1.04 mV, −35.69 ± 1.26 mV, −35.31 ± 0.90 mV, −37.05 ± 2.32 mV and −36.25 ± 1.03 mV, for 3-MT, HVA, β-PEA and 4-AM-1-OH, GABA alone, −35.07 ± 0.90 mV, Supplementary Fig. S2).

**Figure 3:**
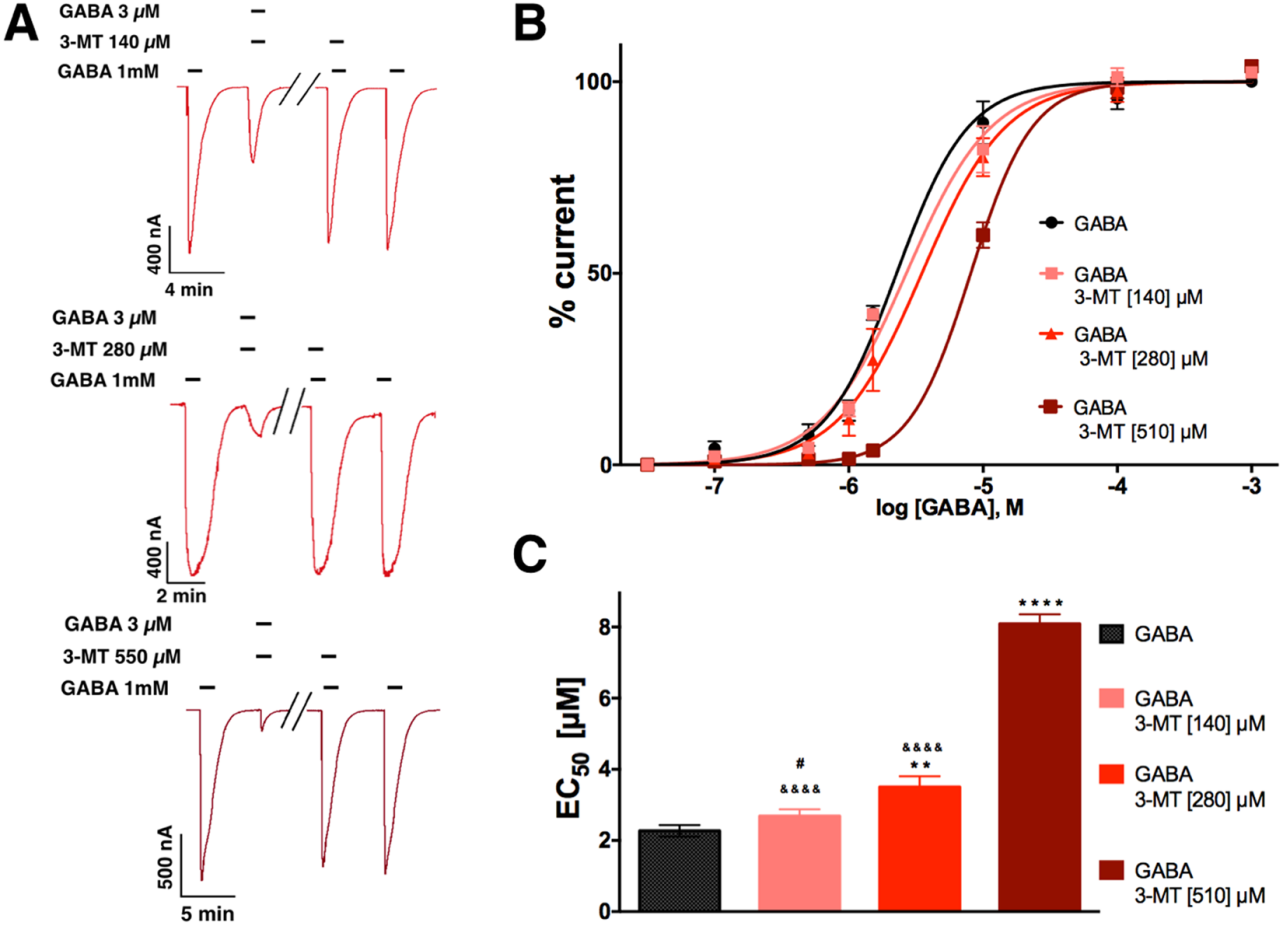
Competition assays. (**A**) Sample currents generate by co-application of GABA (100 nM to 1 mM) and 3-MT (140, 280 and 510 µM), and (**B**) concentration−response relation. Data were normalized to the response to 1 mM GABA. (**C)** Effect of 3-MT concentration of GABA EC_5**0**_. *Significant difference (P value) versus GABA alone, **0.0076, ****<0.0001; ^&^significant difference (P value) versus GABA/3-MT [510] µM, ^**&&&&**^ **<**0.0001; ^**#**^significant difference (P value) versus GABA/3-MT [280] µM, ^**#**^0.0496. Data are from to 6 oocytes from 2 frogs.

Concentration-response curves for the competition assays between 3-MT and GABA were performed in presence of three different concentrations of 3-MT (IC_25_, IC_50_ and IC_75_; Fig. 3A). These showed a shift to the right of the curves as the concentration of 3-MT increased (Fig. 3B), without significant change in the Hill number (1.38 ± 0.24 at IC_25_, 1.40 ± 0.27 at IC_50_ and 1.90 ± 0.36 at IC_75_); these values are statistically non-significant as compared to GABA alone (1.34 ± 0.37). The GABA EC_50_ (2.27 ± 0.40 µM) was higher as the concentration of 3-MT increased; IC_25_ = 2.68 ± 0.4; IC_50_ = 3.50 ± 0.74; and IC_75_ = 8.09 ± 0.65. β-PEA was also evaluated and the results are similar to those found for 3-MT and are shown in Supplementary Figs S3 and S4.

**Figure 4:**
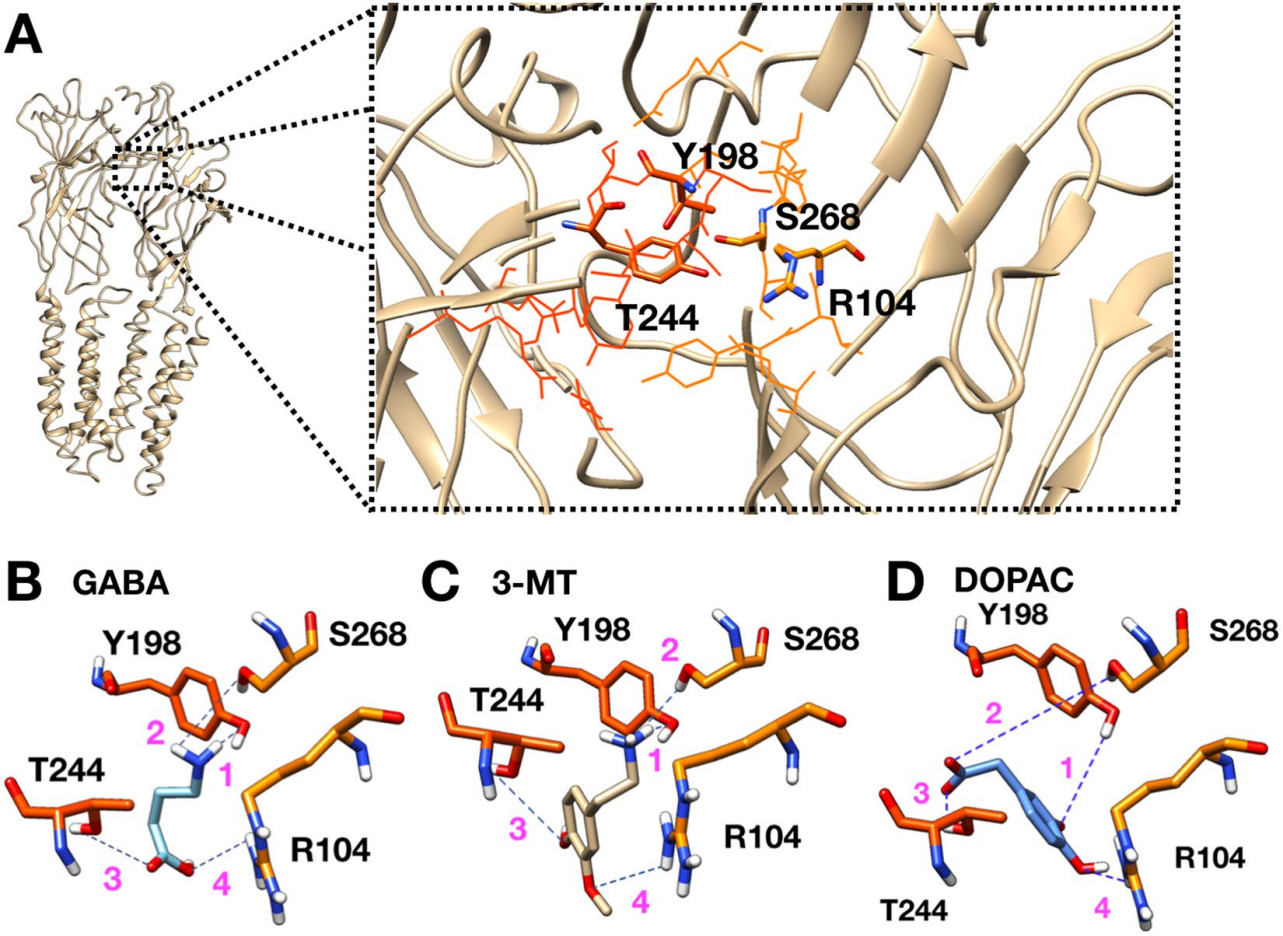
Docking model for GABA and antagonist. (**A)** Structural model of GABAρ1. For clarity, only two of the five subunits are shown. Inset: Enlargement of the agonist pocket formed by 20 residues (wire and stick). Residues from + subunit are in orange and residues from - subunit are in light orange. The key residues Y198, T244, R104 and S268 are represented in stick. (**B**) GABA docked into the key residues and the calculated distances **1** = 2.18, **2** = 3.17, **3** = 3.55 and **4** = 4.27 Å. (**C**) 3-MT docked into the key residues and the calculated distances **1** = 3.06, **2** = 4.18, **3** = 7.78 and **4** = 8.20 Å. The last two distances are too long for establishing a hydrogen bond. (**D)** DOPAC docked into the key residues and the calculated distances **1** = 7.50, **2** = 7.24, **3** = 3.48 and **4** = 4.98 Å. The first two distances are too long for establishing a hydrogen bond. The result suggests a different way for 3-MT and DOPAC anchorage.

The results obtained in the competition assays led us to explore in a molecular docking model the possible ways in which the competitive antagonists bind to the GABA site. For these tests, the GABAρ1 structural model described by Limon *et al*.^29^ was used as a template to evaluate 3-MT and DOPAC. The GABA pocket is immersed within the inter-subunit interface. It is made up of 20 residues that are directly or indirectly involved in GABA binding^30^; two of these residues, located in one of each subunit (Y198 in the + subunit and S168 in the − subunit), are involved in the recognition of the amine group of GABA. The acidic group of GABA is bound to R104^31^ and T244^32^ (in the − and + subunit, respectively) (Fig. 4A). Figure 4 shows the GABA binding pocket occupied by GABA at distances no larger than 6 Å, where the amino moiety of GABA is at 2.18 and 3.17 Å from Y198 and S168, respectively; whereas the distances from the GABA acidic region to T244 and R104 are 3.35 and 4.27 Å, respectively (Fig. 4B). Similar observations were previously reported by Harrison and Lummis^33^. 3-MT fitted the GABA binding site and potentialy binds to Y198 (3.1 Å), S168 (3.2 Å), T244 (7.8 Å) and R104 (6.4 Å) (Fig. 4C). DOPAC also fitted inside the GABA binding site interacting with the same residues: Y198 (7.5 Å), S168 (7.2 Å), T244 (3.48 Å) and R104 (4.98 Å) (Fig. 4D).

**Figure 5:**
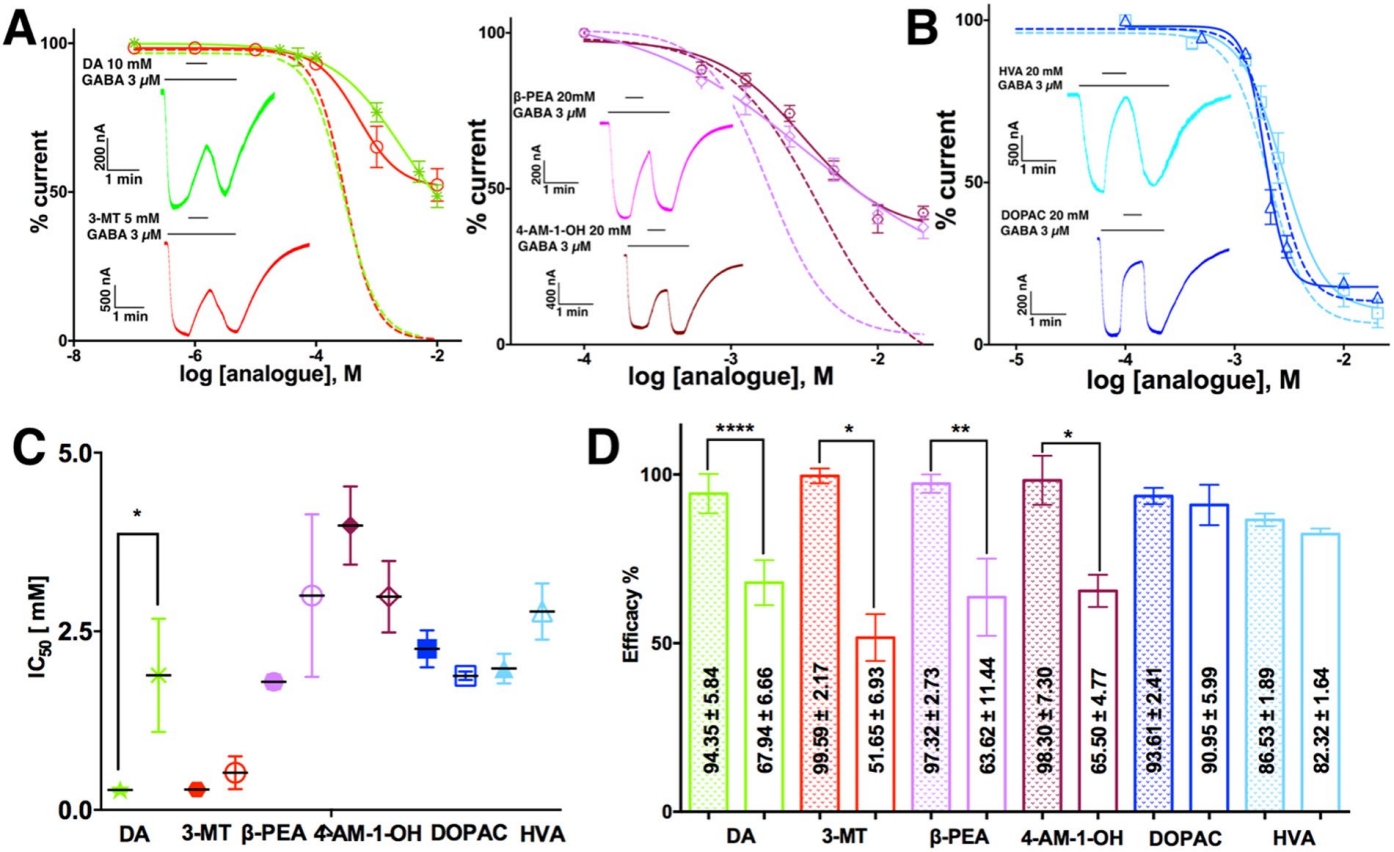
Modulation of GABA responses by DA analogues on activated GABAρ1. (**A**) Sample currents generated by applying the maximal concentrations of DA analogues with an amine moiety on activated receptors (insets) and their corresponding concentration-response curve (continuous line), and concentration-response curve of structural analogous during co-application of GABA (dotted line, from Fig. 1C). Data were normalized to the response to 3 µM GABA. (**B**) Sample currents generated by applying the maximal concentrations of DA analogues with acidic moiety on activated receptors (insets) and their corresponding concentration-response curve (continuous line), and concentration-response curve for analogues during co-application of GABA (dotted line, from Fig. 1C). (**C**) IC_50_ for each analogue when co-applied with GABA perfusion or previously activated receptor. Only DA showed a significant difference between protocols (*). (**D**) Comparison of efficacies between the two protocols (filled bars: same time application, empty bars: activated GABA channels). Molecules that include an amine moiety show significant differences (*). Data in B, D and F are from to 6 oocytes from 3 frogs.

We tested whether DA and its analogues modulated GABAρ1 while maintaining the receptor activated with GABA 3 µM; co-application of DA analogues during GABA perfusion are shown in the representative traces (Fig. 5A,B). All compounds maintained the negative modulation on activated GABAρ1. Molecules with amine moieties showed a tendency to decrease their potency (3-MT and β-PEA), but not in a statistically significant manner since the decay was only 2-fold. However, DA showed the most significant decrease, around 10-fold, whereas the potency of 4-AM-1-OH increased by about 25% and was not statistically significant (Fig. 5C). Molecules with an acidic moiety did not show changes in their potency (Fig. 5B, Eq. 3). Efficacy to inhibit GABA responses under the co-application protocol was considerably lower for DA, 3-MT, β-PEA and 4AM–1-OH, which decreased to 61.94 ± 6.66, 51.65 ± 6.93, 63.62 ± 11.44 and 65.50 ± 4.77%, respectively (all changes are significantly different; Fig. 5A,D).

**Figure 6:**
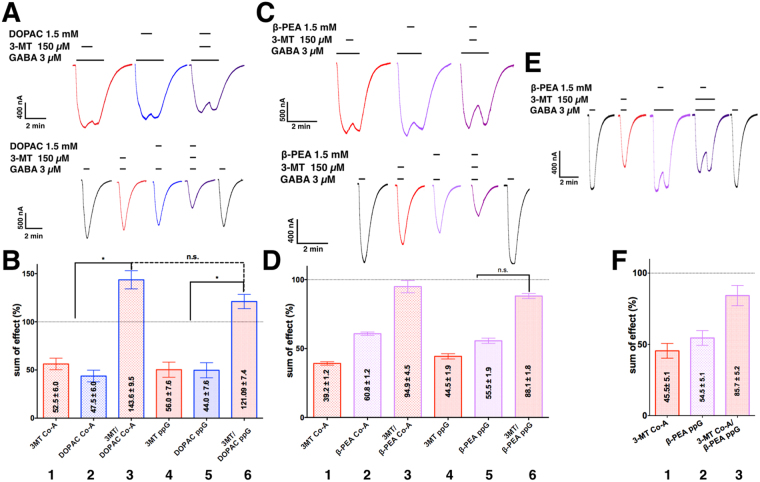
Construction of isobolograms to explore inhibition mechanism. Sample currents of co-applications of 3 µM GABA plus an analogue [at the IC_25_ for each compound and the mix 3 µM GABA/Analogue1[IC_25_]/Analogue2[IC_25_] in (**A**,**C** and **E**,**A**) 3-MT/GABA and /GABA and 3-MT//GABA. (**B**) Normalized responses (bars 1, 2, 4 and 5), experimental responses to the application of the GABA//3-MT mix. Calculated values (bars 3 and 6). *Significant difference versus theoretical value (P = 0.019, 0.046, co-application and prior to perfusion of GABA). (**C**) GABA/3-MT and GABA/β-PEA and mix GABA/3-MT/β-PEA. (**D**) Response normalized to the sum of the individual effects (bars 1, 2, 4), observed responses to the application of the GABA/β-PEA/3-MT mix, the response was normalized to the theoretical value (calculated values in bars 3 and 6, left-right). (**E**) 3-MT/GABA and mix GABA/3-MT/β-PEA. (**F**) Response was normalized to the sum of the individual effects (3-MT for co-application and β-PEA for pre-application of GABA, bars 1 and 2, respectively) on the experimental response to the application of the GABA/3-MT/β-PEA mix. Bar 3, response normalized to the calculated values (data in B, D and F are shown with mean ± S.E.M. Data are from 6 oocytes).

These results suggest a different binding site or inhibitory mechanism that depends on the presence of an amine or acid moiety in the antagonist. To determine whether the binding sites or action mechanism are the same for the open and closed state of the receptor we constructed isobolograms^34^ in three experimental conditions: GABA/analogue1, GABA/analogue2, and GABA/analogue1/analogue2. The theoretical value was obtained from the addition of the individual effects of each analogue in the same oocyte and compared to the effect found in the experimental condition of the mix of both analogues in the same cell. The isobolograms were derived from assays of comparing competency between: (1) GABA and 3-MT and (2) GABA, 3-MT and β-PEA. Figure 6(A,C and E) shows representative traces of the recordings from this series of assays. The first part revealed that the inhibition by the combination 150 µM 3-MT and 1.5 mM DOPAC is more potent than the individual effects of each molecule when the receptor is activated or not (Fig. 6A and B).

In the second part we assessed the interactions between 3-MT/GABA, β-PEA/GABA and 3-MT/β-PEA/GABA. Representative recordings from oocytes expressing GABAρ1 and exposed to 3 µM GABA either alone or with 150 µM 3-MT and/or 1.5 mM β-PEA are shown in Fig. 6C and E.

In contrast to the effect of DOPAC, the addition of β-PEA did not inhibit or potentiate the effect of 3-MT on GABA responses, either when the molecules were perfused all together with GABA or when the receptor was first activated by 3 µM GABA. Figure 6D and F summarizes the results described above.

**Figure 7:**
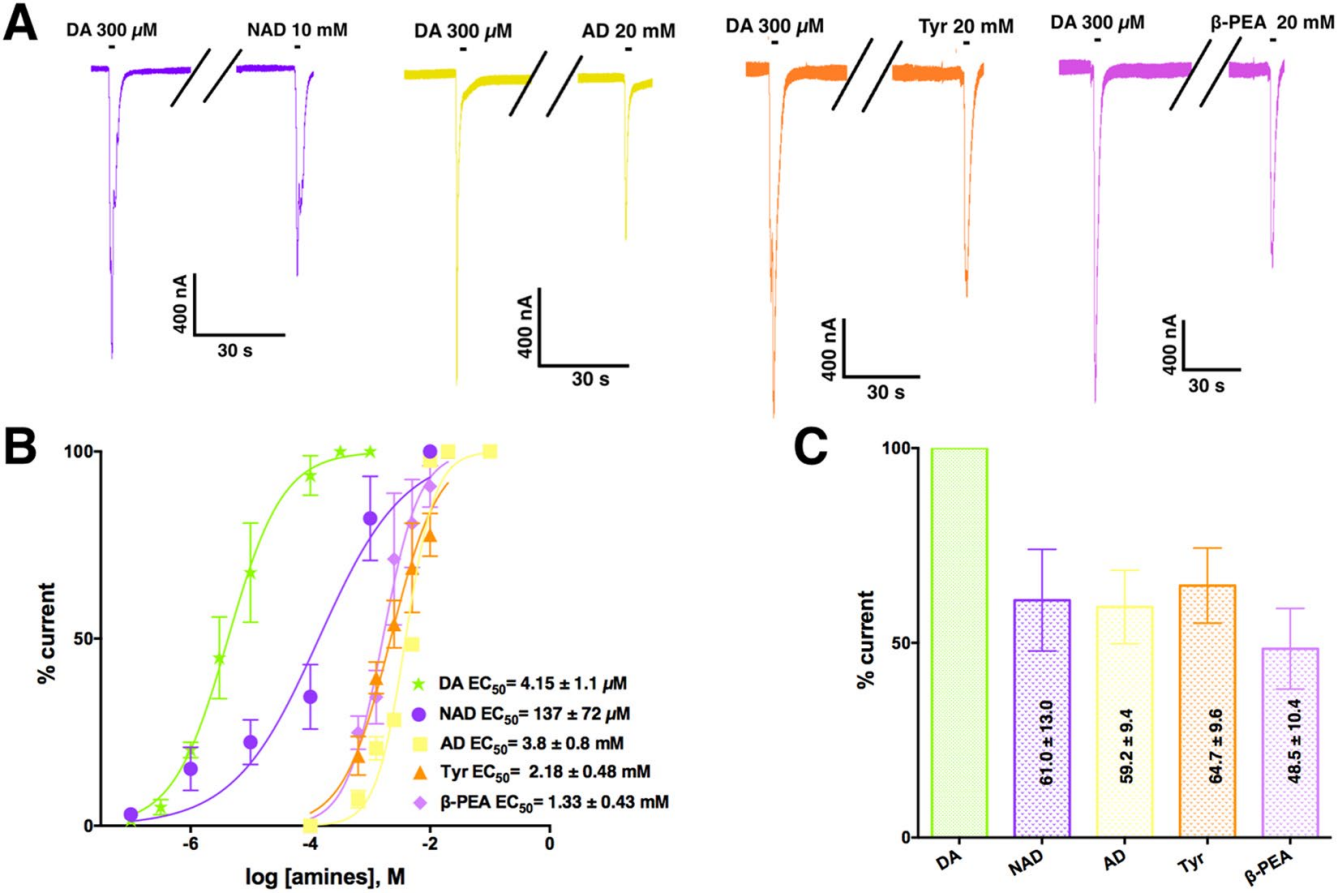
Effect of DA and analogues on new receptor (LGC-53 from *C. elegans*). (**A**) Sample currents generated by DA (300 µM, maximal effect) and other biogenic amines (maximal concentration tested, maximal effect). (**B**) Concentration-response curves of DA and other biogenic amines. (**C**) Maximal effect of the biogenic amine versus maximal response to DA. Data in B and C correspond to the mean ± S.E.M. from 5 oocytes.

Finally, to determine whether the DA analogues activate an ionotropic DA receptor, and thus infer if the agonist-binding site fits these molecules, we expressed LGC-53 in frog oocytes. LGC-53 gates a chloride channel that inactivates promptly and exhibits little or no desensitization after consecutive applications of DA^21^. Neither GABA nor 3-MT gated the channel or showed an evident modulatory effect even at high concentrations (up to 1 mM). DA EC_50_ (4.15 ± 1.1 µM) was similar to the previously reported^21^, whereas NAD activated the receptor with an EC_50_ = 139 ± 35 µM and was more potent than other biogenic amines such as β-PEA (1.63 ± 0.15 mM), Tyr (2.22 ± 0.16 mM) and AD (3.8 ± 0.8 mM, Fig. 7B). None of these amines acted like a full agonist^27^ (Fig. 7A and C). Figure 7A shows sample recordings of oocytes expressing LGC-53 and exposed to several DA analogues. Figure 7B plots the corresponding concentration-response relations, and Fig. 7C compares the maximal effect for each DA analogue.

## Discussion

The results shown here indicate that there may be some interactions between DA and DA metabolites with GABAρ1. In frog oocytes, the effect of DA metabolites on GABAρ1 was concentration-dependent and voltage-independent. These observations extend those described in earlier papers^15,16^ and other evidence that showed that GABA-A receptors are gated by DA in the striatum and other expression systems^17^. Adding to these observations, it was previously demonstrated that DA indirectly modulates GABAρ receptors in isolated cone horizontal cells of catfish^35^ and at the bipolar cell terminals of tiger salamander retina^36^. These findings provided strong evidence for DA modulation of GABA receptor function in the nervous system; however it remains to be explored whether the direct and indirect DA activation/modulation of ionotropic GABA receptors depends on the different subunit combinations that form the receptor in the retina and striatum. Given the present evidence this should be regarded only as a tentative explanation. On the other hand, the role of GABAρ in neural inhibition is well known in retina^13^ and other areas of the nervous system, in which GABA and DA are co-realeased^1^ and where DA can reach concentrations of up to 1.6 mM in synaptic cleft^37^. Our observations were consistent in multiple experiments in different frog donors and RNA preparations, however the question remains whether the metabolites of DA modulate GABAρ1 *in vivo*.

Compared to the other DA metabolites evaluated here, 3-MT turned out to be the most potent modulator of GABAρ1, with a similar potency to DA, suggesting that the methyl moiety in 3-MT does not interfere in ligand binding and may even be important for selectivity of the molecule on ρ1, as described by Krall^38^ with imidazolic core antagonists. Therefore, it is possible that the substitution of the catechol core by a benzodioxol could maintain the effect of DA on the ρ1 receptor as well as the selectivity of 3-MT.

The compounds that showed a modulatory effect on GABAρ1 can be chemically grouped into those that include an amine (3-MT, β-PEA and 4-AM-1OH) or an acidic group (DOPAC and HVA). Since GABA contains both moieties, which interact with the binding site in the receptor, it is feasible for the three compounds to elicit some effect on the receptor. Although none of the tested molecules gate the ion channel of GABAρ1, these three molecules bind to the receptor when it is already in the open conformation; thus, their effect is explained by the structural differences that fluctuate during the cycle of activation-inactivation of GABAρ1. Clear differences in the modulation were observed when DA and DA metabolites were applied after exposing GABAρ1 to GABA. In this protocol, 3-MT showed the largest difference in efficacy as compared to the rest of the compounds.

On the other hand, the structure of HVA and DOPAC includes an acidic moiety and they did not show differences in the efficacy or potency. This is particularly interesting since two other GABAρ1 antagonists with an acidic moiety, (±) −4-ACPAM and SR-95813, show the same effect^39^. DA and 3-MT showed differences in their effect when applied while the receptor was activated or not. This effect is similar to that observed for ginkgolides at GABAρ1. It has been suggested that ginkgolides have two binding sites which are selectively exposed depending on the conformational state of the receptor, thus conferring different affinities for the open and closed states of the channel^40^.

The competition assays indicated that 3-MT, β-PEA and DOPAC share the same binding site with GABA; however, the isobolograms and docking suggested two different mechanisms for inhibition as explained below:

1. Steric impediment. The 3-MT (antagonist, amine) anchored to the GABA binding site prevents the binding of GABA and the activation of GABAρ1. The residues (Y198, S168) described by Sedelnikova^30^ are responsible for the recognition of the amine moiety of GABA. The S168A mutation in GABAρ1 generates functional receptors but 300 less sensitive to the agonist^31^, suggesting that this site is involved in the stabilization of the agonist. Docking modeling shows that 3-MT potentially interacts with the S168 and Y198 residues (at about 5 Å), whereas distances to R104 and T244 residues (involved in channel opening) are considerably larger (more than 7 Å). However, when the agonist-receptor complex is established, 3-MT has a low efficacy to displace the GABA from its binding site.
2. Reduced efficacy by weak interactions. The effect of DOPAC is dependent on the presence of GABA; however, DOPAC does not fully inhibit the GABA currents generated on GABAρ1. This may be explained if the receptor reaches a reduced state of conductivity. Previous reports showed that residues T224 and R104 are critical for interacting with the acidic moiety of GABA and channel gating. Mutations in R104 induce a reconfiguration of the GABA binding site unable to gate the channel^32^, whereas mutation T224A reduces the efficacy of GABA^33^. In our docking model, DOPAC is less than 5 Å from T224 and R104, but the larger distance to Y198 and S168 (more than 7 Å) would make the hydrogen bonds unstable. This structural conformation would be associated to a reduced efficacy and/or a weak interaction with the GABA binding site, thus explaining the remaining current at high concentrations of DA analogues.

On the other hand, it was unexpected to find the modulation of GABAρ1 by 4-AM-1-OH. This can be explained given the similarity with the monoamines and the GABA, in addition the comparison of the alcohol with the agonist suggests the importance of the keto group for the process of opening the channel

Concerning the ionotropic LGC-53 receptor, it is highly selective to DA and does not generate ionic currents when exposed to high concentrations of 5-HT, octopamine and histamine^21,41^. Moreover, we found that it is not modulated by GABA (data not show). We also found that NAD activated LGC-53 with an EC_50_ of 137 µM; thus, this molecule may well gate the ion channel of LGC-53 *in vivo*, whereas AD, Tyr and β-PEA were found to be partial agonists and activated the receptor at high concentrations. Certain interesting possibilities arise in connection with the potential of NAD to activate LGC-53. It might be expected that this biogenic amine interacts with the dopaminergic regulation in *C. elegans*, despite the fact that a noradrenergic system has not been identified in this nematode, it has been observed that NAD induced an oscillatory chloride current in frog oocytes injected with mRNA isolated from *C. elegans* ^42^.

In this work, we show the effect of DA metabolites on GABAρ1 and LGC-53 receptors expressed in *X*. *laevis* oocytes (Table 1). In GABAρ1, 3-MT showed potency and efficacy similar to that of DA, and in terms of potency, the effects of 3-MT and β-PEA do not depend on the state of receptor activation. This contrasts with the effect of DA, which exhibits lower affinity when the receptor channel is open, and with the higher affinity of 4-AM-OH when the GABAρ1 channel is open. In LGC-53, NAD was found to be a full agonist, whereas AD, Tyr and β-PEA are partial agonists. GABA neither activated LGC-53 nor modulated the DA responses. All this evidence suggests: (1) that the human GABAρ1 receptor is inhibited by DA and some DA metabolites, which may be important in areas of the brain where the GABAeric and dopaminergic systems converge; and (2) that the nematode ionotropic receptor LGC-53 is activated by DA analogues but not activated by GABA.

**Table 1:** Effect of DA and metabolites on GABAρ1 and LGC-53 receptors.

Compound	Structure	RECEPTOR (LGIC super family)			
		GABA ρ*1* (*Human*)		LGC-53	
		IC_50_ (mM)	Antagonist: Full or partial	EC_50_ (mM)	Agonist: Full or partial
Dopamine (DA)*		0.210 ± 0.00112	full	0.0042 ± 0.001	full
3- Methoxytyramine (3-MT)		0.285 ± 0.003	full	N.E	**
***4-Amino-1-butanol*** (***4-AM-1OH***)		3.91 ± 0.544	full	N.E	**
3,4-Dihydroxyphenylacetic acid (DOPAC)		2.25 ± 0.258	partial	N.E	**
Homovanillic acid (HVA)		1.97 ± 0.211	partial	N.E	**
2-Phenylethylamine (***β-PEA***)		1.79 ± 0.104	full	1.33 ± 0.433	partial
Tyramine (Tyr)*		0.55 ± 0.012	full	2.18 ±0.482	partial
Noradrenaline (NAD)		N.E	**	0.137 ± 0.072	partial
Adrenaline (AD)		N.E	**	3.8 ± 0.803	partial
L-3,4-dihydroxyphenylalanine (L-DOPA)		N.E	**	N.E	**

*Data from Ochoa de la Paz *et al*., 2012; N.E., no effect, **not applicable.

## Material and Methods

### Expression of GABAρ1 and LGC-53 receptors in *X. laevis* oocytes

All frogs were handled in accordance with the guidelines of the National Institute of Health Guide for Care and Use of Laboratory Animals and with the approval of the Institutional Animal Care and Use Committee of the National University of Mexico. Frogs were anesthetized, and follicles were removed manually and treated with 0.3 µg/µl collagenase type I in Ca^2+^-free Ringer’s at room temperature for 30 min. Oocytes were maintained at 16 °C in Barth’s medium: 88 mM NaCl, 1 mM KCl, 0.33 mM Ca_2_(NO)_3_, 0.41 mM CaCl_2_, 0.82 mM MgSO_4_, 2.4 mM NaHCO_3_, 5 mM HEPES, pH 7.4, and 0.1 mg/mL gentamycin sulfate. The next day, 50 nL (1 µg /µL) of human GABAρ1 mRNA (cDNA encoding in pcDNA3 plasmid^43^ or LGC53 mRNA^21^ (for *in vitro* transcription, we used the mMessage mMachine kit) were injected into each oocyte, and the electrophysiological recordings were obtained 1–5 days after injection.

### Voltage clamp recordings

Membrane currents produced by the agonists were recorded using the two-microelectrode voltage-clamp technique^42–44^ with an AXOCLAMP-2B amplifier (Axon Instruments) and DIGIDATA 1440 A (Molecular Devices) and pClamp 10.5 software (Molecular Devices). Oocytes were placed in a recording chamber of 0.5 mL and impaled with two glass microelectrodes filled with 3 M KCl, with resistances in the range of 0.5 to 2.0 MΩ, and the membrane potential was held at −60 mV. All recordings were done at room temperature. Oocytes were continually perfused (20 mL/min) with Ringer’s solution: 115 mM NaCl, 2 mM KCl, 1.8 mM CaCl_2_, and 5 mM HEPES, pH 7.4. All compounds were purchased from Sigma-Aldrich, GABA, A5835-25G; DA, PHR1090-1G; 3-MT, 4251-100MG; DOPAC, 850217-1 G; HVA, H1252-1G; 4-AM-1-OH, 178330; β-PEA, P6513-25G; AD, E4250-500MG; NAD, A7257-500MG; Tyr, T-7255 and L-DOPA, D-9628-5G. Aliquots of 1 M GABA were stored at −30 °C. DA and analogues were prepared in Ringer’s solution right before application. The pH of all solutions was adjusted to 7.4. All compounds were applied in bath solution. To determine the concnetration-response relation, the agonist (GABA 3 µM) was applied before and after the mix of GABA 3 µM/analogue to evaluate the ability of DA and analogues to modulate GABAρ1 while the receptor was activated. The analogue was applied during the current plateau upon activation with 3 µM GABA. To activate the LGC-53 receptor and determine its modulation by DA analogues, DA was applied before and after the analogue.

### Data analysis

All data were analyzed using GraphPad Prism 6.0 software (GraphPad Software Inc., San Diego CA) and expressed as mean ± S.E.M. Each data point was obtained from to 6 cells from at least 3 frogs. The effects of dopamine analogues were evaluated by comparing the response obtained by the application of 3 μM GABA, and the inhibition was determined as follows:

1 $${\rm{Inh}}[{\rm{Ana}}]{\rm{i}}={{\rm{2I}}}_{[{\rm{Ana}}]{\rm{i}}}/({{\rm{I}}}_{[\mathrm{GABA\; 3}\mu M]{\rm{pre}}}+{{\rm{I}}}_{[\mathrm{GABA}3\mu M]\mathrm{post}})$$

where, Ihn [Ana]: % of inhibition by the analogue at concentration i when co-applied with 3 μM GABA; I_[Ana]i_: current obtained by the co-application of the analogue at concentration i and 3 μM GABA; I_[GABA 3μM] pre_: current obtained by 3 μM GABA before the co-application; I_[GABA 3μM]post_: current obtained by 3 μM GABA after the co-application. Concentration-response relations were plotted according to equation:

2 $${\rm{Y}}=100/(1+{10}^{\wedge }(({{\rm{LogIC}}}_{{\rm{50}}}-{\rm{X}})\,\ast \,\mathrm{nH}))$$

where X is the logarithm of analogue concentrations, IC_50_ is the antagonist concentration ([Ana]) causing half-maximal inhibition of GABA, and nH is the Hill coefficient. When complete inhibition was not obtained, the following equation was applied:

3 $${\rm{Y}}={{\rm{I}}}_{{\rm{\min }}}+({{\rm{I}}}_{{\rm{\max }}-}{{\rm{I}}}_{{\rm{\min }}})/(1+({\rm{X}}/{\rm{IC50}}))$$

where I_min_ represents the residual GABA current remaining with a maximal concentration of analogue and I_max_ current obtained by 3 μM GABA. IC_50_ is the concentration of antagonist that gives a response halfway between Bottom and Top.

### Molecular Docking

We used the homology model described by Lima *et al*.^29^, the ligands were optimized with Avogadro^45^, and the docking was done with Autodock vina 4.2^46^ for visualization of the solutions and measurement of distances used USFC Chimera^47^.

Significant differences between groups were determined by a *t*-test for unpaired two-tailed groups. Significant differences were P: ns >0.05 >* >0005 >** >0.0008 >*** <0.0001.

## Electronic supplementary material

Supplemetary figures
