# Supplementary material for: Antagonistic effect of dopamine structural analogues on human GABAρ1 receptor
Source: Sci Rep. 2017 Dec 12;7:17385. doi: 10.1038/s41598-017-17530-8 (PMC5727059; doi:10.1038/s41598-017-17530-8)
Supplement: Supplementary file 1 — Supplemetary figures [file 41598_2017_17530_MOESM1_ESM.pdf]

Antagonistic effect of dopamine structural analogues on human GABAρ1 receptor.

Alfredo Alaniz-Palacios and Ataulfo Martínez-Torres*

Departamento de Neurobiología Celular y Molecular, Laboratorio de Neurobiología Molecular y Celular, Instituto de Neurobiología, Universidad Nacional Autónoma de México, Juriquilla, 76230 Santiago de Querétaro, Querétaro, México

*Corresponding author. Tel.: +52 442 238 1064, fax: +52 442 238 1064. E-mail address: ataulfo@unam.mx (A. Martínez-Torres).

Keywords: LGC53, Ligand-gated ion channels, monoamines, neurotransmitter receptors, GABA Modulators

Supplementary figure S1

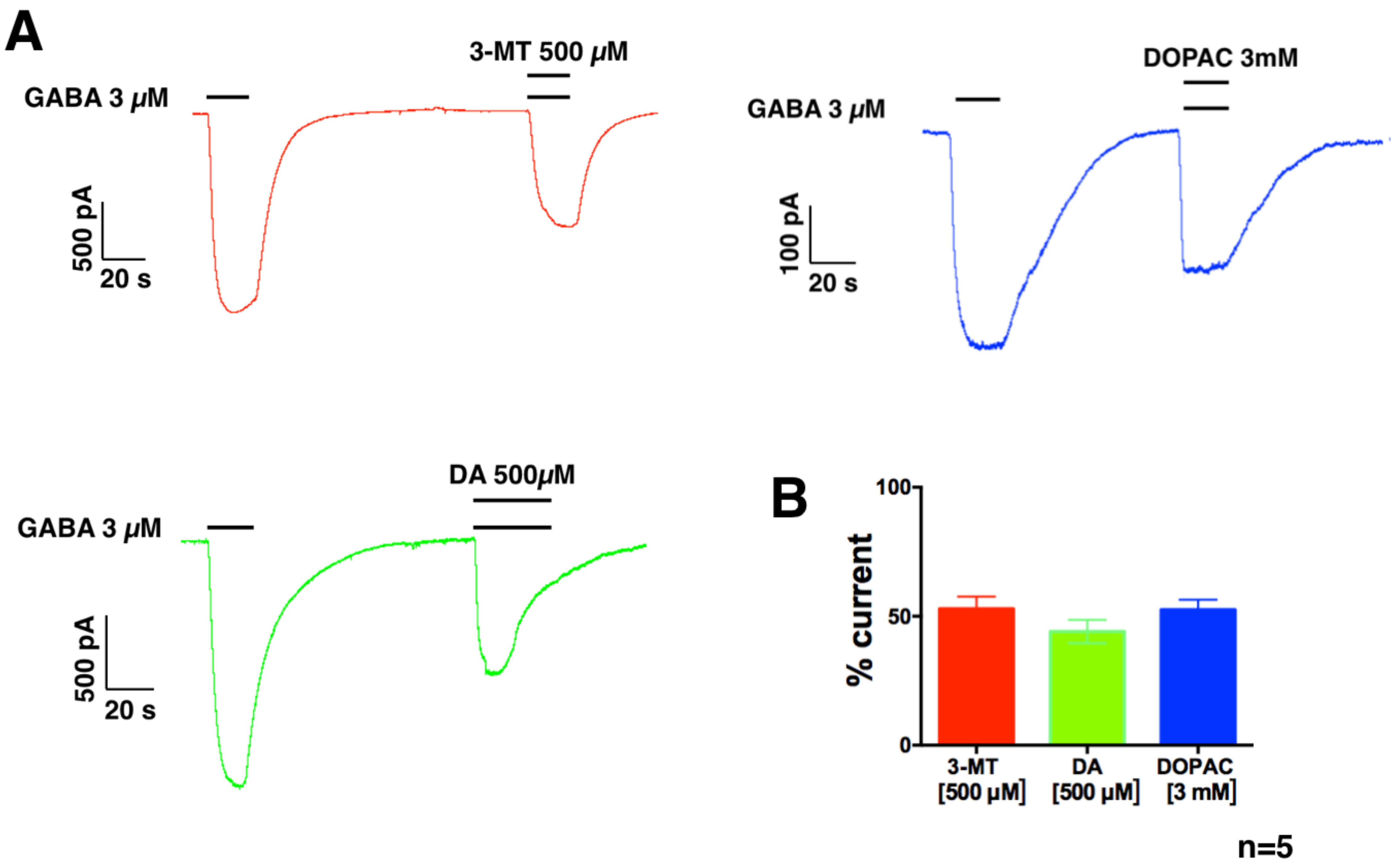

Supplementary Figure S1. Antagonistic effect of DA analogues on ρ1 expressed in HEK cells. **A)** Sample currents of co-applications of 3 μM GABA and 3-MT (500 μM), DA (500 μM) and DOPAC (3 mM). **B)** Percentage of inhibition for DA and DA analogues in the current evoked by 3 μM GABA.

Supplementary figure S2

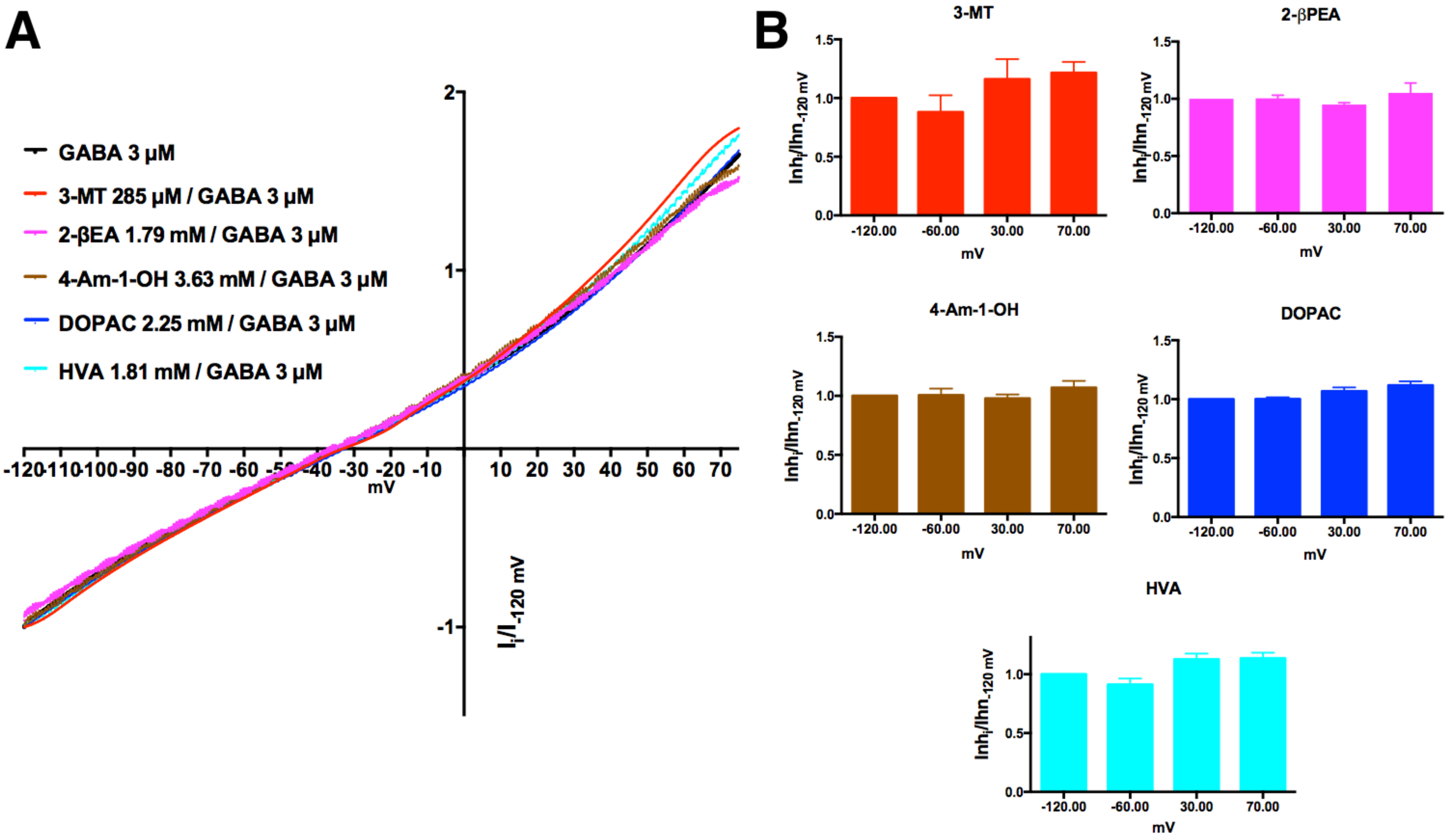

Supplementary Figure S2. I/V Curves A) Current–voltage relations for GABA currents modulated in presence of DA analogues. **B)**. Percentage of inhibition of DA analogues (for each compound the IC_{50}s were tested) in the currents generated by 3 μM GABA, plotted at –120, –60, +30 and + 70 mV. Reverse potentials were: -35.25 ± 1.04 mV, -35.69 ± 1.26 mV, -35.31 ± 0.90 mV, 37.05 mV and 36.25 mV for 3-MT, HVA, β-PEA and 4-AM-1-OH, respectively. GABA reverse potential was -35.07 ± 0.90 mV.

Supplementary figure 3

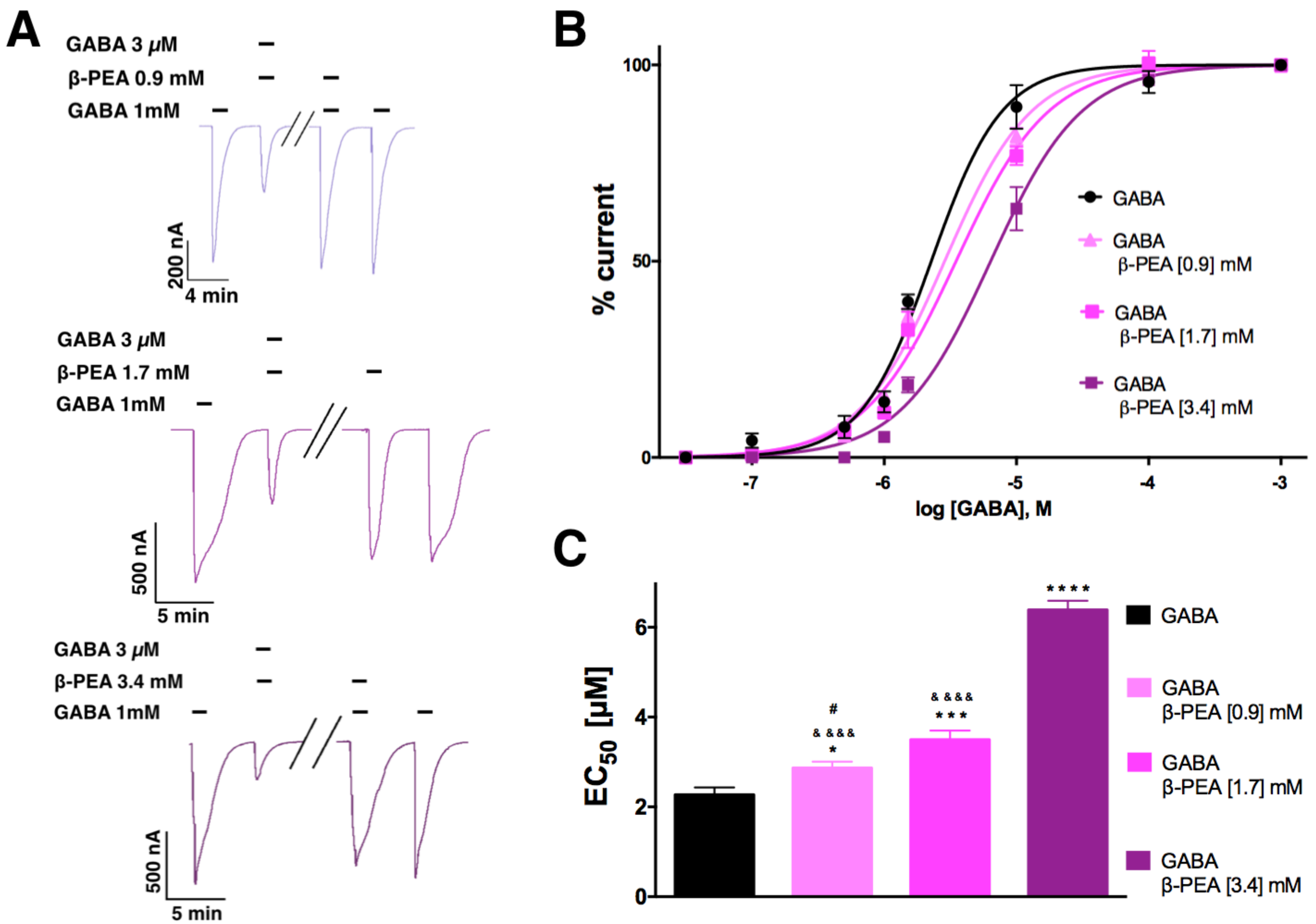

Supplementary Figure S3. Competition assays. **A)** Sample currents for co-application assay of GABA (100 nM to 1 mM) and β-PEA (0.9, 1.7 and 3.4 mM), and **B)** concentration–response curve. Data were normalized to the response to 1 mM GABA. The loss of the antagonistic effect by β-PEA at high concentrations suggests competition. **C)** Effect of DA analogues on GABA EC_{50}; * significant difference (P value) versus GABA alone, * = 0.02, *** = 0.0009, **** < 0.0001; **&** significant difference (P value) versus GABA / β-PEA [3.4] mM **&&&&** < 0.0001, **#** significant difference (P value) versus GABA / β-PEA [1.7] mM, **#** = 0.032. Data from to 6 oocytes from 2 frogs.

Supplementary figure 4

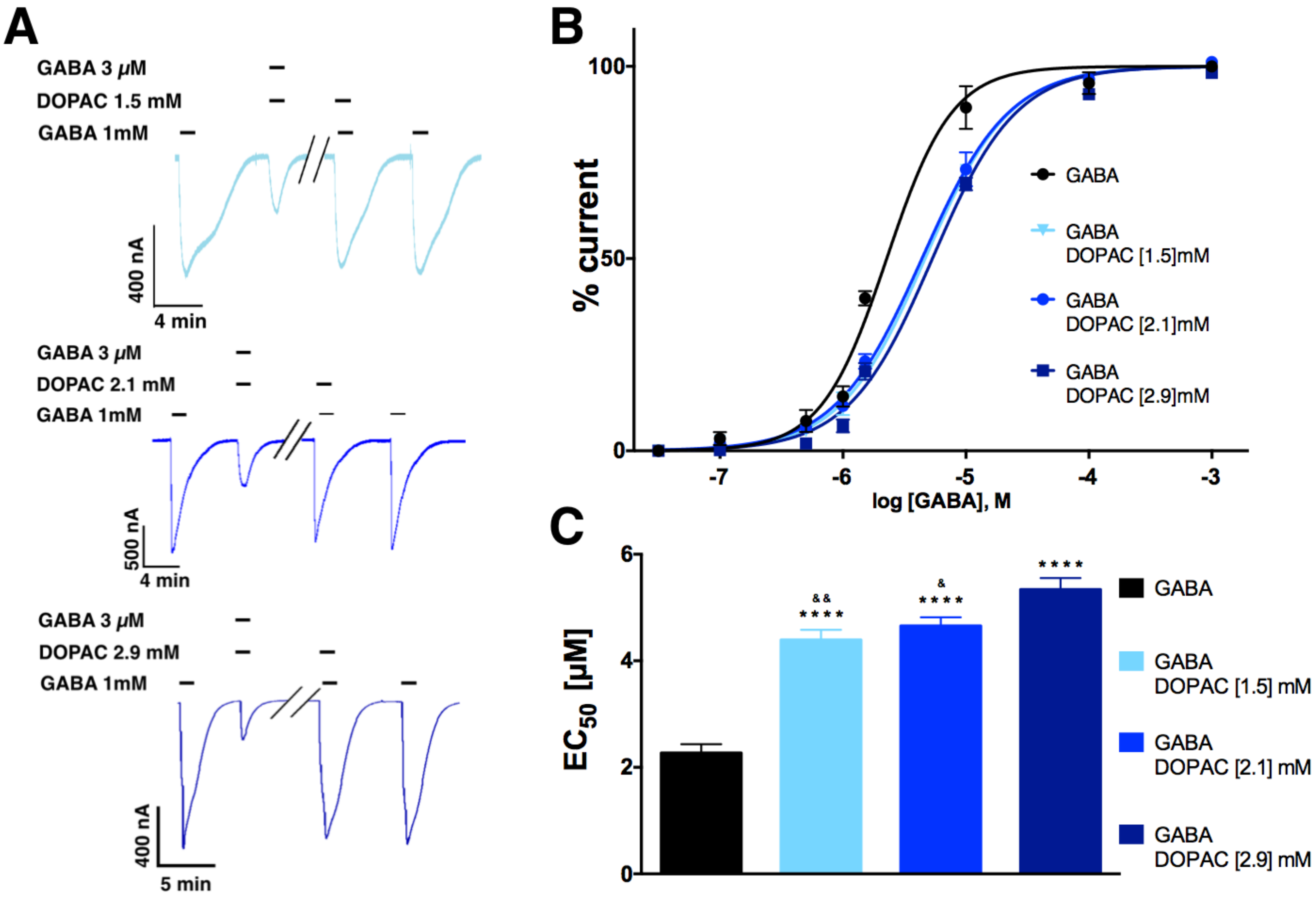

Supplementary Figure S4. Competition assays. **A)** Sample currents of co-application of GABA (100 nM to 1 mM) and DOPAC (1.5, 2.1 and 2.9 μM) and, **B)** concentration–response curve. Data were normalized to the response to 1 mM GABA. The loss of the antagonistic effect by 3-MT for increase of the agonist suggests a competitive mechanism. **C)** Changes in EC_{50} of GABA; * significant difference (P value) versus GABA alone, ** = 0.0076, ****< 0.0001; **&** significant difference (P value) versus GABA / 3-MT [510] μM, **&&&&** < 0.0001; **#** significant difference (P value) versus GABA / 3-MT [280] μM, **#** = 0.0496. Data are from to 6 oocytes from 2 frogs.
